# Effect of Quercetin on Lipids Metabolism Through Modulating the Gut Microbial and AMPK/PPAR Signaling Pathway in Broilers

**DOI:** 10.3389/fcell.2021.616219

**Published:** 2021-02-09

**Authors:** Mi Wang, Bo Wang, Shanshan Wang, Han Lu, Hao Wu, Manyi Ding, Linlin Ying, Yanjun Mao, Yao Li

**Affiliations:** ^1^Institute of Animal Nutrition, Northeast Agricultural University, Harbin, China; ^2^College of Animal Husbandry and Veterinary Medicine, Jinzhou Medical University, Jinzhou, China

**Keywords:** quercetin, lipids metabolism, microbial, AMPK, PPAR

## Abstract

The present study was conducted to investigate effects and mechanism of quercetin on lipids metabolism in broilers. 480 AA broilers were randomly allotted to four treatments (0, 0.2, 0.4, and 0.6 g/kg quercetin) for 42 days. Compared with the control, 0.6 g/kg quercetin significantly decreased percentage of abdominal fat (*P* < 0.05); 0.2, 0.4, and 0.6 g/kg quercetin significantly decreased relative abundance of *Lachnospiraceae* and *Desulfovibrionaceae* (*P* < 0.05, *P* < 0.05, *P* < 0.01; *P* < 0.01, *P* < 0.01, *P* < 0.01); 0.2 g/kg quercetin significantly increased mRNA expression of PI3K, AMPKα1, AMPKα2, AMPKβ2, LKB1 (*P* < 0.01, *P* < 0.01, *P* < 0.05, *P* < 0.01, *P* < 0.05), and significantly reduced mRNA expression of SREBP1 and PPARγ (*P* < 0.01, *P* < 0.05); 0.4 g/kg quercetin significantly increased mRNA expression of LKB1 and PKB (*P* < 0.05, *P* < 0.01) and significantly reduced mRNA expression of ACC, HMGR, PPARγ, and SREBP1 (*P* < 0.05, *P* < 0.01, *P* < 0.01, *P* < 0.01); 0.6 g/kg quercetin significantly increased mRNA expression of AMPKγ, LKB1, CPT1, PPARα, PKB (*P* < 0.01, *P* < 0.01, *P* < 0.01, *P* < 0.05, *P* < 0.05), and significantly reduced the mRNA expression of PI3K, ACC, HMGR, PPARγ, SREBP1 (*P* < 0.05, *P* < 0.05, *P* < 0.01, *P* < 0.01, *P* < 0.01); 0.2 g/kg quercetin significantly increased protein expression of AMPK (*P* < 0.01); 0.6 g/kg quercetin significantly increased protein expression of LKB1 (*P* < 0.01), 0.2 and 0.6 g/kg quercetin significantly increased protein expression of PI3K, PKB, CPT1 (*P* < 0.05, *P* < 0.01, *P* < 0.05, *P* < 0.01, *P* < 0.01, *P* < 0.01), and significantly reduced protein expression of ACC and SREBP1 (*P* < 0.01, *P* < 0.01, *P* < 0.01, *P* < 0.01). In conclusion, quercetin improved lipid metabolism by modulating gut microbial and AMPK/PPAR signaling pathway in broilers.

## Introduction

Abdominal fat is essentially excessive accumulation of lipid. Therefore, the research of lipid metabolism becomes the focus at present. Lipid metabolism has also been linked with differences in the composition of the gut microbiota (Huazano-Garcia et al., 2017). In high-fat diet (HFD) fed Wistar rats, an increase in abundance of the families *Coriobacteriaceae* and *Enterobacteriaceae* was reported that may directly alter host physiology (Lecomte et al., 2015). The previous results of transcriptome sequencing showed that adenosine monophosphate activated protein kinase (AMPK) signaling pathway was the main signal pathway of lipid metabolism (Wang et al., 2020). Activated AMPK pathway reduced lipid synthesis by suppressing the expression of downstream targets (Zhao et al., 2019). Moreover, activation of PPAR pathway significantly alleviated lipid metabolic disorders (Cai et al., 2020). PPAR is an AMPK downstream target (Diniz et al., 2021), AMPK/PPAR signaling pathway was the main signal pathway of lipid metabolism.

Plant polyphenol, especially flavonoids, is a kind of safe additives with multiple biological activities. It drew public attention because of anti-bacterial action, anti-inflammation, anti-cancer and immune enhancement, etc. (George et al., 2016; Goya et al., 2017). Quercetin (International Union of Pure and Applied Chemistry nomenclature for quercetin is 3,3′,4′,5,7-pentahydroxyflvanone), a flavonoid found in fruits and vegetables, is categorized as a flavonol, which is one of the six subclasses of flavonoid compounds (Li et al., 2016a, b). Quercetin supplementation also improved antibacterial capacity, antioxidation and lipid metabolism in broilers (Sohaib et al., 2015). The previous studies in our laboratory showed that quercetin improved immune function and antibacterial activities in broilers (Wang et al., 2018; Yang et al., 2020). However, the percentage of abdominal fat was the most important indicator of carcass characteristics in broilers, the objective of this study was to investigate the mechanism of quercetin on lipid metabolism in broilers.

## Materials and Methods

### Birds, Diets, and Experimental Design

All procedures were performed in accordance with the guidelines set forth by the Animal Welfare Committee of Northeast Agricultural University (Harbin, China). Housing, management and care of the birds confirmed to the guidelines of Agricultural Animal in Agricultural Research and Teaching of Heilongjiang Province (HEI Animal Management Certificate No. 11928).

Four hundred and eighty AA broilers (1 day old) were obtained from a commercial facility (Yinong Poultry, Harbin, China). Birds were randomly allotted to four experimental treatments comprising six replicates of 20 birds in each replicate. All birds were raised in stainless steel cages (316 mm × 400 mm × 400 mm) under continuous light in a controlled room for 42 days. The room temperature was maintained at 33°C for the first 3 days. Then the temperature was reduced to 24°C until the end of the experiment. Water and experimental diets were provided *ad libitum*.

The experimental diets were based on corn and soybean meal, and quercetin was added at four concentrations: 0, 0.2, 0.4, and 0.6 g/kg of diet. Feeding was divided into two phases: the starter from 1 to 21 days and the grower from 22 to 42 days. The basal diet was formulated to meet the nutritional requirements suggested according to Chinese Broiler Feeding Standards (NY/T33-2004) (Table 1). Quercetin (purity of quercetin dihydrate powder ≥97%, Sigma-Aldrich, St. Louis, MO, United States) was mixed in basal diet.

**TABLE 1:** Calculated composition of basal diets and nutrient level.

	% (Air-dry basis)	
Composition	1–21 days	21–42 days
Ingredients		
Corn	57.50	62.30
Soybean meal	34.50	30.00
Fish meal	1.00	1.00
Soybean oil	3.00	3.00
Sodium chloride	0.30	0.30
Dicalcium phosphate	1.65	1.70
Limestone	1.52	1.17
Methionine	0.20	0.20
Choline	0.10	0.10
Multivitamin premix^a^	0.03	0.03
Mineral premix^b^	0.20	0.20
Nutrient level		
Metabolizable energy (MJ/kg)	12.33	12.50
Crude protein	21.75	19.72
Lysine	1.18	1.04
Methionine + Cysteine	0.91	0.86
Ca	1.07	0.60
Total *P*	0.70	0.68
Available *P*	0.46	0.45

*^*a*^Content per kilogram of diet: 1,500 IU of vitamin A; 3,200 IU of vitamin D3; 10 IU of vitamin E, 0.5 mg of vitamin K, 1.8 mg of vitamin B1, 3.6 mg of vitamin B2, 3.5 mg of vitamin B6, 0.01 mg of vitamin B12, 0.15 mg of biotin, 0.55 mg of folic acid, 30 mg of niacin, and 10 mg of pantothenic acid. ^*b*^Content: 8 mg of Cu (CuSO_4_⋅5H_2_O), 0.35 mg of I (KI), 80 mg of Fe (FeSO_4_⋅7H_2_O), 60 mg of Mn (MnSO_4_⋅H_2_O), 0.15 mg of Se (NaSeO_3_), and 40 mg of Zn (ZnO).*

### Methods

#### Carcass Characteristics

At the age of 42 days, 12 chickens per treatment (6 per replicate pen) of randomly chosen were slaughtered for carcass analyses. Each of these birds was deprived of feed for 12 h and individually weighed just prior to slaughter. Percentage of carcass, eviscerated and semi-eviscerated, breast muscle, thigh muscle and abdominal fat was calculated according to the weight of the carcass, eviscerated, semi-eviscerated, breast muscle, thigh muscle, and abdominal fat.

#### Metagenome Sequencing

The whole ileal contents were collected and frozen in liquid nitrogen and sent to Geneis (Beijing) Co. Ltd. for metagenome sequencing using Illumina HiSeq 2500 platform. Microbial DNA was extracted from 12 ileum samples using the improved metagenomic DNA extraction method. The quality of the extracted metagenomic DNA was checked on 0.8% agarose gel visualized on a gel documentation system (Alphaimager HP, United States). The quantity and purity of the DNA was assessed using Nanodrop LITE spectrophotometer (Thermo Scientific, United States). ABI Steponeplus Real-Time PCR System and Agilent 2100 Bioanalyze were used to detect the output and quality of the constructed library.

#### AMPK Signaling Pathway

##### Real-time quantitative PCR (RT-qPCR)

Liver tissue was individually homogenized, and total RNA was extracted using the TRIZOL reagent. The Superscript First-Strand Synthesis System (Life Technologies, Grand Island, NY, United States) was adopted to synthesize first-strand cDNA from the total RNA. The quantity of purified cDNAs was determined by RT-qPCR (Life Technologies, Grand Island, NY, United States). β-actin was used as the internal control in this study (Table 2).

**TABLE 2:** Parameters of primer pairs for the genes.

Gene	Primer sequence	Product size	GenBank accession
PPARα	F: 5′-TAACGGAGTTCCAATCGC-3′	222 bp	NM 001001464
	R: 5′-AACCCTTACAACCTTCACAA-3′		
PPARγ	F: 5′-CACTGCAGGAACAGAACAAAGAA-3′	67 bp	NM 001001460
	R: 5′-TCCACAGAGCGAAACTGACATC-3′		
PKB	F: 5′-CTGATGATGCCAAGGAGATT-3′	175 bp	NM 205055.1
	R: 5′-TGGTCAGGAGGAGTGATTGT-3′		
AMPKα1	F: 5′-AAGGTTGGCAAGCATGAGTT-3′	492 bp	NM 001039603
	R: 5′-TTCTGGGCCTGCATATAACC-3′		
AMPKα2	F: 5′-AGCACGCCAACAGAGACTTCTT-3′	399 bp	NM 001039605
	R: 5′-ATCATCAAAGGGCAAAGTGC-3′		
AMPKβ1	F: 5′-CCAGGAGCCCTATGTCTGTAAG-3′	113 bp	NM 001039912
	R: 5′-CAGGATCGCAAGAAATGCC-3′		
AMPKβ2	F: 5′-GCACTGCCCATCCCTCTAA-3′	125 bp	NM 001044662
	R: 5′-CGGCTGCCACGAAACAA-3′		
AMPKγ	F: 5′-AGAGGTCCCAAAGCCTGAGTT-3′	118 bp	NM 001034827
	R: 5′-GAAGATGCCCAGAGCCACA-3′		
PI3K	F: 5′-CGGATGTTGCCTTACGGTTGT-3′	162 bp	NM 001004410.1
	R: 5′-GTTCTTGTCCTTGAGCCACTGAT-3′		
ACC	F: 5′-CACTTCGAGGCGAAAAACTC-3′	447 bp	NM 205505
	R: 5′-GGAGCAAATCCATGACCACT-3′		
CPT1	F: 5′-CAATGAGGTACTCCCTGAAA-3′	337 bp	NM oo102898
	R: 5′-CATTATTGGTCCACGCCCTC-3′		
HMGCR	F: 5′-AGCTGCAACCCTGAGGAAACT-3′	1268 bp	AB109635
	R: 5′-AGCCATCACTGTAGCACACAC-3′		
SREBP1	F: 5′-GAGGAAGGCCATCGAGTCA-3′	392 bp	AY 029224
	R: 5′-GGAAGACAAAGGCACAGAGG-3′		
LKB1	F: 5′-GGGGAGACAGAAGGGAACAGA-3′	158 bp	NM 001045833
	R:5′-TGAGAGGGATGCTTGAATACGA-3′		
β-actin	F: 5′-TGCGTGACATCAAGGAGAAG-3′	300 bp	L08165
	R: 5′-TGCCAGGGTACATTGTGGTA-3′		
18sRNA	F:5′-TAGATAACCTCGAGCCGATCGCA-3′	312 bp	AF 173612
	R:5′-GACTTGCCCTCCAATGGATCC TC-3′		

##### Western blot

Briefly, equal amounts of protein samples (30 μg) were loaded into SDS-PAGE apparatus and transferred to PVDF membranes. PVDF membranes were then probed with primary antibodies against targeted proteins. Images were detected by a ChemiDoc XRS + imaging system (Bio-Rad, Hercules, CA, United States), and bands of the target proteins were quantified with the ImageJ software. GAPDH was used as internal control in this study.

### Statistical Analysis

The data was treated using a one-way analysis of variance as a completely randomized design with four treatments and six replicates for each treatment using SPSS 20.0 statistical software, the results were expressed as means ± standard error of the mean (SEM), *P* < 0.05 was considered as statistically significant criteria. Calculated Δ*Ct* (corrected sample) = mean value of target gene–mean value of internal reference gene, ΔΔ*Ct* = Δ*Ct* − mean value of control group. The results of western blot were analyzed by Gel-Pro analyzer 4 software.

## Results and Discussion

### Effect of Quercetin on Carcass Characteristics in Broilers

There are few reports on the effects of dietary quercetin supplementation on the carcass characteristics in broilers. 0.2% sea buckthorn flavonoid supplementation significantly increased the dressing percentage, improved eviscerated and semi-eviscerated percentage in AA broilers (Li et al., 2008). However, our study showed that no significant differences in percentage of dressing, eviscerated weight, semi-eviscerated of AA broilers were observed (*P* > 0.05), compared with control (Table 3). The difference of carcass characteristics probably resulted from complicated constituent of flavonoids from sea buckthorn, and/or diverse bioavailability and synergism of various flavonoids (Ross and Kasum, 2002; Guo and Bruno, 2015).

**TABLE 3:** Effects of dietary quercetin on carcass characteristics in AA broilers (%).

	Diet (quercetin, g/kg)			
Items	0	0.2	0.4	0.6
Dressing percentage	94.41 ± 1.15	94.20 ± 1.42	93.10 ± 3.99	90.89 ± 6.63
Eviscerated percentage	69.86 ± 3.17	71.51 ± 2.03	69.49 ± 4.69	69.32 ± 3.61
Semi-eviscerated percentage	85.48 ± 2.51	86.09 ± 1.55	84.72 ± 4.37	84.97 ± 3.91
Percentage of abdominal fat	1.53 ± 0.23^a^	1.48 ± 0.20^a^	1.33 ± 0.15^ab^	1.19 ± 0.26^b^

*In the same row, values with different small letter superscripts mean significant difference (*P* < 0.05); Values with different capital letter superscripts mean significant difference (*P* < 0.01); Values with no letter or the same letter superscripts mean no significant difference (*P* > 0.05). Values are expressed as means ± SEM, and *n* = 6 for all groups. The same as the follows.*

Some studies had shown that quercetin promoted fat metabolism in rats (Peng et al., 2017; Rocca et al., 2018). Abdominal fat deposition was reduced by Hawthorn extract in the drinking water of chickens (Ahmadipour et al., 2014). Kim reported that high intake of dietary flavonoids may be associated with a decreased prevalence of abdominal obesity in broilers (Cao et al., 2012). In the present study, the percentage of abdominal fat was significantly decreased by 0.6 g/kg quercetin supplementation (*P* < 0.05) (Table 3). The result was supported by the findings which fermented *Ginkgo biloba* leaves (including abundant flavonoid) in the diet of broilers decreased abdominal fat deposition in adults (Seong-Ah et al., 2020). Lipid accumulation may contribute to abdominal fat, therefor, accumulation of lipids could be attributed to the downregulation of fatty-acid oxidation and adipogenic and lipogenic pathways upregulation and increased delivery of fatty acid to abdominal (Foulds et al., 2017).

### Effect of Quercetin on Relative Abundance of Ileal Microflora in AA Broilers at the Family

**FIGURE 1:**
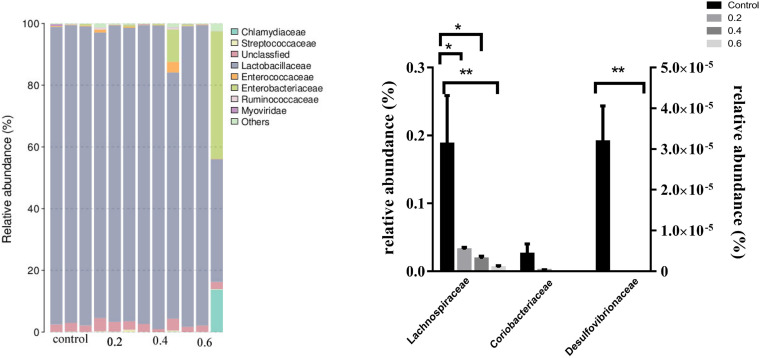
Effect of quercetin on relative abundance of ileal microflora in AA broilers at the family level. Note: The results of relative quantification were expressed as 2^– ΔΔCT^. The quantification of control was 1, namely 2^– ΔΔCT^ = 1. The value 2^– ΔΔCT^ of treatment group was a multiple of control. *N* = 3. ^∗^*P* < 0.05, ^∗∗^*P* < 0.01. Values are mean ± SEM (*n* = 3).

Accumulating evidence indicates that microorganisms improved host physiology and lipid metabolism. Metagenomic sequencing technology is widely used in microflora detection. The *Desulfovibrionaceae* family (*Proteobacteria* phyla) is gram-negative sulfate-reducing bacteria involved in the production of lipopolysaccharides and endotoxins and well-known inflammation-inducing capacity (Cani et al., 2008; Zhao L. et al., 2017). *Desulfovibrionaceae* was thought to be positively associated with obesity (Delzenne and Cani, 2011), the metagenomic analyses showed that the tea extracts changed the overall composition of gut microbiota and decreased the relative abundance of family *Rikenellaceae* and *Desulfovibrionaceae* in mice (Liu J. et al., 2019). Green tea polyphenol (epigallocatechin-3-gallate-EGCG) significantly decreased the relative abundance of *Desulfovibrionaceae* in HFD fed mice (Ushiroda et al., 2019). The abundance of bacterial genera *Desulfovibrionaceae* was significantly decreased in Wasabi-treated rats (Thomaz et al., 2020). Moreover, the relative abundance of *Desulfovibrionaceae* in the HFD group was significantly higher than that in the ND group, however, after chlorogenic acid treatment, the relative abundance of this bacteria was decreased in mice (Wang et al., 2019). *Lachnospiraceae*, a family of *clostridia*, a kind of digestive tract-associated bacteria, correlates with increased fat mass and lipid level (Kameyama and Itoh, 2014; Murugesan et al., 2016; Pataky et al., 2016). *Lachnospiraceae* family was accompanied with the increasing body weight in germ-free *ob/ob* mice (Zhao L. et al., 2017). Previous studies have shown that *Lachnospiraceae* may protect against obesity and colon cancer in humans by producing butyric acid (Meehan and Beiko, 2014). Additionally, *Lachnospiraceae* was largely decreased when HFD-induced mice were simultaneously administrated chlorogenic acid (Wang et al., 2019). Furthermore, high dose of Fuzhuan brick tea (FBT) reduced the levels of *Lachnospiraceae* and *Desulfovibrionaceae*, compared with the baseline level, the beneficial effects on HFD-induced obese mice were associated with regulating the relative abundance of *Lachnospiraceae* and *Desulfovibrionaceae* (Liu D. et al., 2019). *Coriobacteriaceae* family in the gut was associated with the development of metabolic syndrome (Luccia et al., 2015). *Coriobacteriaceae* belonging to the phylum *Actinobacteria* were involved in bile acid metabolism, had a negative effect on cholesterol homeostasis through increasing cholesterol absorption (Zhao N. Q. et al., 2017). Specific species within *Coriobacteriaceae* were known for metabolizing compounds such as the isoflavones daidzein and genistein to equol (Bangsgaard Bendtsen et al., 2012; Flórez et al., 2019). Equol may significantly affect blood lipids *in vitro* (Zhang T. et al., 2013). In the current study, at the family level, 0.2, 0.4 and 0.6 g/kg quercetin supplementation significantly reduced the relative abundance of *Lachnospiraceae* and *Desulfovibrionaceae* (*P* < 0.05, *P* < 0.05, *P* < 0.01; *P* < 0.01, *P* < 0.01, *P* < 0.01); However, 0.2, 0.4, and 0.6 g/kg quercetin supplementation did not influence the relative abundance of *Coriobacteriaceae* (*P* > 0.05) (Figure 1). Together with the above results, we inferred that quercetin reduced percentage of abdominal fat through beneficial modulation of the gut microbiota.

### Quercetin Improved Lipids Metabolism Through AMPK/PPAR Signal Pathway in Broilers

**FIGURE 2:**
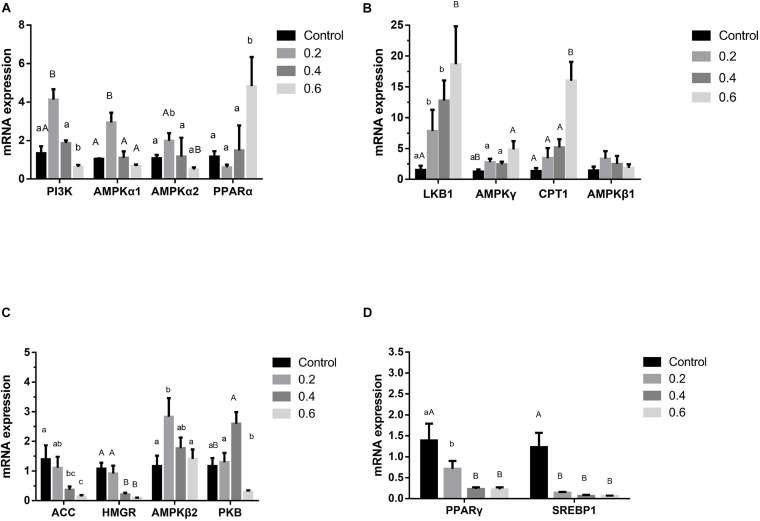
Effects of quercetin on genes relating to the AMPK/PPARα signaling pathway in liver of AA broilers. Note: The results of relative quantification were expressed as 2^– ΔΔCT^. The quantification of control was 1, namely 2^– ΔΔCT^ = 1. The value 2^– ΔΔCT^ of treatment group was a multiple of control. *N* = 6. Mean values without a common letter are significantly different, *P* < 0.05. Values are mean ± SEM (*n* = 6).

**FIGURE 3:**
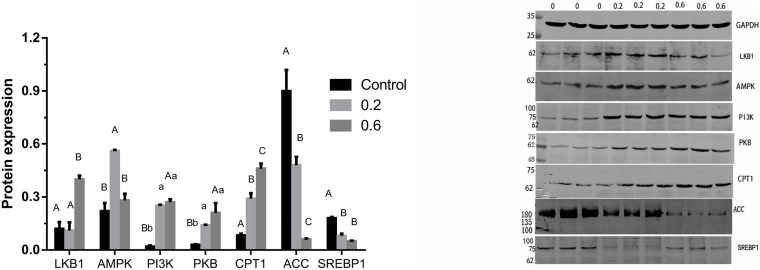
Effects of quercetin on protein relating to the AMPK/PPARα signaling pathway in liver of AA broilers. Note: Data are presented as mean ± SEM (*n* = 3–5). Bars with different lowercase letters are significantly different (*P* < 0.05) Bars with different capital letters are significantly different (*P* < 0.01).

The previous study in our lab found 505 differentially expressed genes of AMPK signal pathway in the quercetin treatment, compared with the control, and the liver was the main metabolic site of lipid in broilers (Wang et al., 2020); 0.04% quercetin supplementation decreased fat content of liver in laying hens (Zhang L. et al., 2013). Therefore, these findings together with the present study confirmed that quercetin regulated fat metabolism, thus reduced abdominal fat deposition in broilers. However, the mechanism of action remains to be unclear. AMPK plays a key role in regulating lipid and glucose metabolism, acts as an energy sensor, regularly responding to cellular energy demands by sensing the balance in AMP to ATP ratio (Zhao N. Q. et al., 2017). AMPK, a heterotrimer kinase composed of catalytic and regulatory subunit, is classified into three different receptor subtypes, AMPKα, AMPKβ, and AMPKγ. AMPK signaling pathway coordinates glucose metabolism by regulating glycolysis and gluconeogenesis, and controls lipid metabolism by acting on fatty acid synthesis and fatty acid oxidation (Hardie, 2011; Do et al., 2012). Some studies suggested that the enhanced AMPK signaling may attenuate liver lipid accumulation and hepatic fibrosis in mice (Wang et al., 2013; Woods et al., 2017). Phosphorylated AMPK level was down-regulated in the diabetic liver, and *Sonchus oleraceus* Linn increased the expression of AMPK in diabetes mice (Chen et al., 2020). Ginsenoside Rk3 (G-Rk3) mediated hepatic lipid accumulation via activating the AMPK/Akt signaling pathway in mice (Liu Y. et al., 2019). Licochalcone A activates AMPK to increase lipolysis in liver (Liou et al., 2019). Quercetin exerted anti-adipogenic effects in 3T3-L1 cells by activating the AMPK signaling pathway (Yang et al., 2008). A previous study reported that quercetin increased the phosphorylation of AMPK in cultured smooth muscle cells and aortic arteries, which also exhibited increased levels of acetyl CoA carboxylase, a downstream protein of AMPK, implicating the increased activity of AMPK following quercetin administration (Ahn et al., 2008). In addition, flaxseed polysaccharide interacts with intestinal flora, upregulates AMPK, and inhibited lipid accumulation in obese mice (Luo et al., 2019). In the current study, 0.6 g/kg quercetin supplementation significantly increased AMPKγ mRNA expression (*P* < 0.01). Simultaneously, 0.2 g/kg quercetin supplementation significantly increased mRNA expression of AMPKα1, AMPKα2, AMPKβ2 (*P* < 0.01, *P* < 0.05, *P* < 0.05) (Figure 2) and protein expression of AMPK (*P* < 0.01) (Figure 3). These findings suggested that quercetin regulated lipid metabolism through increasing the expression of AMPK.

#### The AMPK Upstream Pathway

Phosphatidylinositol 3-kinase (PI3K) and serine-threonine protein kinase (PKB/AKT), regarded as signal transduction molecules in cells, are associated with varieties of biological processes, including apoptosis, insulin resistance, and adipogenesis (Maingrette and Renier, 2003; Jiménez-Castro et al., 2013; Pang et al., 2013). Previous studies elucidated that the PI3K-PKB/AKT mediated signaling pathway was participated in lipid accumulation process via phosphorylating or activating substrates (Peng et al., 2015; Zhu et al., 2015; Manning and Toker, 2017). The activated insulin receptor activates PI3K and PKB/AKT as well as downstream glucose and lipid metabolism. In the current study, 0.2 g/kg quercetin supplementation significantly increased mRNA and protein expression of PI3K and protein expression of PKB (*P* < 0.01, *P* < 0.05); 0.6 g/kg quercetin supplementation significantly increased protein expression of PI3K and PKB (*P* < 0.01, *P* < 0.01) (Figures 2, 3). Taken together, in line with the previous research that quercetin improved lipid metabolism and reduced abdominal fat deposition by activating PI3K/PKB signal pathway (Ying et al., 2020). And together with the results of AMPK in this experiment, our findings indicated that quercetin increased the expression of AMPK via stimulating PI3K-PKB/AKT kinase activity.

Liver kinase B1 (LKB1) is a serine/threonine protein kinase which was first discovered in studying Peutz-Jeghers syndrome. AMPK activity is mainly regulated by LKB1 in mice (Sakamoto et al., 2005) and chickens (Proszkowiec-Weglarz et al., 2006). LKB1 regulation of the AMPK family plays well-established roles in increasing fat oxidation (Thomson et al., 2007), while mediating part of the response to oxidative stress (Chen et al., 2016). LKB1 is upstream of AMPK and a family of 12 other Ser/Thr kinases closely related to AMPK, which would potentially be regulated of fat acid oxidation by LKB1 in mice (Kim et al., 2018). LKB1 is considered the major route of AMPK activation because an LKB1 deficiency results in an almost complete loss of AMPK activity (Jeppesen et al., 2013). Our study results showed that 0.2, 0.4 and 0.6 g/kg quercetin supplementation significantly increased LKB1 mRNA expression (*P* < 0.05, *P* < 0.05, *P* < 0.01), and 0.6 g/kg quercetin supplementation significantly increased protein expression of LKB1 (*P* < 0.01) (Figure 2). Together with the results of AMPK in this experiment, our findings indicated that quercetin-activated LKB1 up-regulated AMPK expression.

#### The AMPK Downstream Pathway

Acetyl-CoA carboxylase (ACC), a rate-limiting enzyme involved in the production of malonyl-CoA, is used for fatty acyl-CoA biosynthesis, stimulates CPT1 and reduces the flux of substrates in the fatty acid anabolic pathway (Carling et al., 2008; Lage et al., 2008). ACC inactivation is related to the predominance of β-oxidation, which provides energy to the body (Zhou et al., 2001). AMPK may regulate the transcription and expression of ACC in hypothalamus of mammals and avian species (Xue and Kahn, 2006; Xu et al., 2011, 2012). Curcumin significantly decreased levels of ACC1 to inhibit lipid metabolism (Qiu et al., 2016). Our study showed that 0.4 and 0.6 g/kg quercetin supplementation significantly reduced ACC mRNA expression (*P* < 0.05, *P* < 0.05); 0.2 and 0.6 g/kg quercetin supplementation significantly reduced protein expression of ACC (*P* < 0.01, *P* < 0.01). Together with the results of AMPK in this experiment, the current results were consistent with Watt MJ’s study which AMPK activation down-regulated ACC expression in liver of broilers (Watt et al., 2006).

Carnitine palmitoyl transterase-1 (CPT1) is considered as a mitochondrial gateway for fatty acid entering into the matrix, is also the main modulator of hepatic mitochondrial β-oxidation flux. CPT1 adjusts the β-oxidation of fatty acids by catalyzing the conversion of fatty acyl-CoA into fatty acylcarnitine in mitochondria (Zheng et al., 2013). Joubert et al. (2010) demonstrated that AMPK may regulate fatty acid metabolism by the CPT in muscle of broilers. Nobiletin increased hepatic CPT1 mRNA, thus promoted fatty acid oxidation (Mulvihill et al., 2011). Instant fermented teas heighten energy expenditure by increasing the expression of the CPT-1 gene (Sun et al., 2019). In the current study, 0.6 g/kg quercetin supplementation significantly increased CPT1 mRNA expression (*P* < 0.01); 0.2 and 0.6 g/kg quercetin supplementation significantly increased protein expression of CPT1 (*P* < 0.01, *P* < 0.01) (Figure 2). Together with the results of AMPK in this experiment, our findings showed that quercetin down-regulated the expression of ACC in liver, indicating that inhibition of ACC by AMPK activation contributed to increased CPT 1 activity.

Peroxisome proliferator activated receptors (PPARs) are nuclear transcription factors, which are classified into three different receptor subtypes, PPARα, PPARβ, and PPARγ. PPARs are particularly expressed in tissues with high lipid catabolic capacities, such as liver, skeletal muscle and brown adipose tissue, play a crucial role in lipid metabolism by regulating oxidation and disintegration of fatty acids, lipid transportation, assembly of lipoproteins through modulating transcription of their downstream genes (Daigo et al., 2007; Park et al., 2014; Beatriz et al., 2017). Berbamine treatment increased the PPARα expression, a vital transcription factor to fatty acid oxidation (Ankita et al., 2020). Meanwhile, previous studies have revealed that AMPK activation is accompanied by increased PPARα expression (Baar, 2004; Lee et al., 2006). Wogonin exhibited beneficial effects in lipid metabolism through activating AMPK and PPARα (Bak et al., 2014). The transcription factor PPARγ plays a key role in regulating adipogenesis and is expressed in the late stages of differentiation. Red yeast buckwheat (RYB) treatment significantly suppressed the mRNA and protein expression of PPARγ in 3T3-L1 cells (Hong et al., 2017). Rosehip extract inhibited lipid accumulation in white adipose tissue by suppressing the expression of PPARγ (Akifumi et al., 2013). Flavonol kaempferol decreased the AMPK activation-mediated PPARγ expression (Zhang and Liu, 2011). Our study results showed that 0.6 g/kg quercetin supplementation significantly increased PPARα mRNA expression (*P* < 0.05), and 0.2, 0.4, and 0.6 g/kg reduced PPARγ mRNA expression (*P* < 0.05, *P* < 0.01, *P* < 0.01) (Figure 2). It suggested that AMPK activation was accompanied by increased PPARα and reduced PPARγ expression. AMPK activation by quercetin in the present study regulated ACC, CPT1, and PPAR expression, thus increased lipid β-oxidation, therefore, decreased fat deposition.

Sterol regulatory element binding proteins (SREBPs) play pivotal roles in both lipogenesis and cholesterol homeostasis (Zhu et al., 2019). SREBP1 is particularly involved in activation of the genes controlling fatty acid metabolism and *de novo* lipogenesis (Hua et al., 2016). SREBP1c manages adipogenesis by activating some genes connected to the synthesis of fatty acids and triglyceride (Li et al., 2011; Hu et al., 2020). Berberine may prevent lipid metabolism disorders by down-regulating SREBP and up-regulating AMPKα (Li et al., 2011). Several studies demonstrated that AMPKα reduced lipid synthesis by restraining SREBP activity and promoted fatty acid oxidation to control hepatic energy metabolism in liver (Park et al., 2008; Li et al., 2011). Our study has shown that 0.2, 0.4, and 0.6 g/kg quercetin supplementation significantly reduced mRNA and protein expression of SREBP1 (*P* < 0.01, *P* < 0.01, *P* < 0.01, *P* < 0.01, *P* < 0.01) (Figures 2, 3). Together with the results of AMPK in this experiment, our findings indicated that quercetin decreased SREBP1 expression thought AMPK activation in liver of broilers.

3-Hydroxy-3-Methylglutaryl-CoA reductase (HMGR) is a rate-limiting enzyme for cholesterol synthesis. Transcriptional and pathway analysis results showed that the overexpression of HMGR was correlated with the down-regulation of AMPK gene expression (Lin et al., 2020). Schisandra chinensisfruit (SF) extract may decrease lipid accumulation by up-regulating lipolytic factors (AMPK) and decreasing the expression or activity of lipogenic modulators (HMGR) (Liu et al., 2015). Artemisia species treatment significantly reduced HMGR and PPARα activation in comparison with high fat diet mice (Wang et al., 2013). In the current study, 0.4 and 0.6 g/kg quercetin supplementation significantly reduced HMGR mRNA expression (*P* < 0.01, *P* < 0.01) (Figure 2). Our findings were supported by Haitao Liu’s study (Liu et al., 2015) that AMPK activation down-regulated HMGR expression in liver of broilers. The fat of meat is an important component in meat quality and impacts animal productivity. Therefore, it suggested that quercetin-activated AMPK in the present study down-regulated HMGR and SREBP1 expression, thus decreased lipid deposition.

**FIGURE 4:**
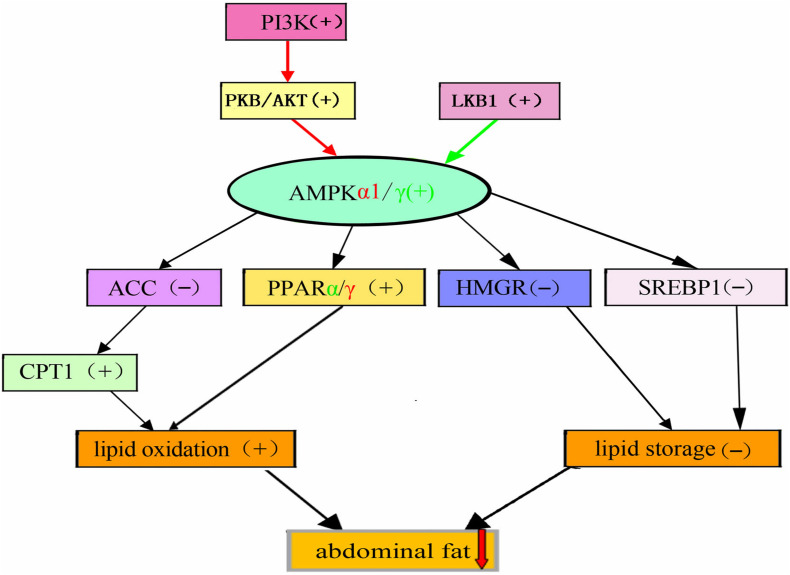
Proposed model of AMPK actions on gene expressions in liver of chickens fed with quercetin [(^–^), Down, (+), Up] change.

Quercetin reduced the expression levels of SREBP1, PPARγ, and HMGR in liver by activating the AMPK signaling pathway. Consequently, adipogenesis was restricted, thereby reduced lipid synthesis. The present study also found that quercetin decreased ACC expression and increased the expression of CPT1 and PPARα by activating AMPK, thus prevented fatty acid intake and promoted lipolysis and fatty acid oxidation. However, quercetin activated the AMPK signaling pathway through increasing the expression of PI3K, PKB/ATK and LKB1. Therefore, the current results demonstrated that dietary quercetin supplementation improved lipid metabolism, which promoted lipid oxidation and reduced lipid deposition by regulating AMPK/PPAR signaling pathway in liver of broilers (Figure 4).

## Conclusion

The present results showed that dietary quercetin supplementation might change the abdominal fat deposition by regulating AMPK/PPAR signaling pathway and gut microbial in broilers. Activation of the AMPK/PPAR signaling pathway and modulation of the gut microbiota attenuated abdominal fat accumulation by accelerating lipolysis and fatty-acid oxidation and inhibiting fatty acid uptake and lipid synthesis, accumulation of lipids could be repression, quercetin may be used as functional additive to improve lipid metabolism.

## Data Availability Statement

The raw data supporting the conclusions of this article will be made available by the authors, without undue reservation.

## Ethics Statement

The animal study was reviewed and approved by the HEI Animal Management Certificate No. 11928.

## Author Contributions

MW participated in the design of the study and critically revised the first manuscript. SW, HL, HW, and LY provided some technical support for the experiment. BW, MD, and YM performed the experiments and participated in the statistical analysis. YL modified the manuscript and have given final approval of the version to be submitted. All authors contributed to the article and approved the submitted version.

## Conflict of Interest

The authors declare that the research was conducted in the absence of any commercial or financial relationships that could be construed as a potential conflict of interest.
